# Carbohydrate intake and cardiometabolic risk factors in high BMI African American children

**DOI:** 10.1186/1743-7075-7-10

**Published:** 2010-02-09

**Authors:** Sushma Sharma, Lindsay S Roberts, Robert H Lustig, Sharon E Fleming

**Affiliations:** 1 The Dr Robert C and Veronica Atkins Center for Weight and Health, University of California, Berkeley, CA 94720-3104, USA; 2 Department of Nutritional Sciences and Toxicology, University of California, Berkeley, CA 94720-3104, USA; 3 Division of Pediatric Endocrinology, University of California, San Francisco, CA 94143, USA

## Abstract

The aim of this study was to evaluate the relationship between intakes of subgroups of energy-providing carbohydrate, and markers of cardiometabolic risk factors in high BMI African American (AA) children.

A cross sectional analysis was performed on data from a sample of 9-11 year old children (n = 95) with BMI greater than the 85^th ^percentile. Fasting hematological and biochemical values for selected markers of cardiometabolic risk factors were related to intakes of carbohydrates and sugars.

After adjusting for gender, pubertal stage and waist circumference, multivariate regression analysis showed that higher intakes of carbohydrate (with fat and protein held constant) were associated with higher plasma concentrations of triglycerides (TG), VLDL-C, IDL-C, and worse insulin resistance (homeostasis model assessment of insulin resistance, HOMA-IR). After dividing carbohydrate into non-sugar versus sugar fractions, sugars were significantly related to higher TG, VLDL-C, IDL-C, lower adipocyte fatty acid insulin sensitivity (ISI-FFA), and was closely associated with increased HOMA-IR. Similar trends were observed for sugars classified as added sugars, and for sugars included in beverages. Further dividing sugar according to the food group from which it was consumed showed that consuming more sugar from the candy/soda food group was highly significantly associated with increased TG, VLDL-C, IDL-C and closely associated with increased HOMA-IR. Sugars consumed in all fruit-containing foods were significantly associated with lower ISI-FFA. Sugars consumed as fruit beverages was significantly associated with VLDL-C, IDL-C and ISI-FFA whereas sugars consumed as fresh, dried and preserved fruits did not show significant associations with these markers.

Sugars consumed from in all dairy foods were significantly associated with higher TG, VLDL-C and IDL-C, and with significantly lower HDL-C and ISI-FFA. These effects were associated with sugars consumed in sweetened dairy products, but not with sugars consumed in unsweetened dairy products. This analysis suggests that increases in carbohydrate energy, especially in the form of sugar, may be detrimental to cardiometabolic health in high BMI children.

## Introduction

Childhood obesity has reached epidemic levels in developed countries [1]. Obesity during childhood and adolescence is associated with a number of cardiometabolic risk factors [2]. Evidence suggests that diet during childhood may have important implications for the development of obesity and chronic disease in later life. Also, it has been established that energy intake from specific macronutrients plays a more important role in the development of obesity and metabolic complications than does total energy intake alone. Our recent findings suggest that the effect of increased energy on risk of developing cardiometabolic risk factors is in part influenced by the source of that energy [2].

The African American (AA) population has been shown to be at higher risk for both obesity and the metabolic syndrome than are Caucasians [3]. As the prevalence of obesity [4] and metabolic syndrome in AA children continues to increase [5], management of risk factors must begin at an early age. Recently, we concluded that increases in energy intake from carbohydrate were associated with undesirable effects, including increases in TG, VLDL-C, IDL-C and HOMA-IR [2]. More recently, increased attention to *type *of carbohydrate, rather than *total *carbohydrate, has begun to clarify the role of carbohydrate subgroups in the obesity epidemic among adults; however studies of this nature have not yet been performed in children. Numerous short-term studies have shown that diets high in carbohydrates, particularly sugars, and even more particularly sucrose and fructose, increase serum TG concentrations and decrease serum HDL cholesterol; and may therefore increase the risk of CVD [6,7]. It is unclear which specific subgroups of carbohydrates are possibly related to cardiometabolic risk factors in AA children. In this study we evaluated the relationship between intakes of different subgroups of carbohydrates, and selective markers of cardiometabolic risk factors in high BMI AA children.

## Methods

### Subjects

Subjects included in this analysis represented a cross-sectional convenience sample of 9-11 year-old African American children enrolled in the *Taking Action Together *study, an inner-city YMCA-based intervention trial that aimed to reduce risk factors for type 2 diabetes. A total of 128 African-American (AA) children (56 boys, 65 girls) were evaluated in this cross-sectional analysis, and a full set of data was available for 99 children.

All participants had BMIs above the 85^th ^percentile, fasting glucose <120 mg/dl, were free from any known metabolic diseases, and were not taking medications known to affect the study outcomes. Parental informed consent was obtained for all subjects, and all protocols were approved by institutional review boards at the University of California, Berkeley and San Francisco. Anthropometric characteristics were measured using procedures previously described [8].

### Biochemical measurements

Subjects reported to Children's Hospital and Research Center, Oakland, CA after a 12 hr overnight fast when their blood was drawn. Plasma lipids (lipoprotein cholesterol and triglycerides) were measured by a comprehensive lipoprotein analysis (VAP-cholesterol method) performed by a commercial lab (Laboratory Corporation of America) as previously described [2]. Fasting concentrations of plasma glucose, serum insulin, and non-esterified fatty acids were measured, and these values used to calculate insulin resistance (HOMA-IR) and adipocyte insulin sensitivity (ISI-FFA) [2,9]. Pubertal development was assessed by measurements of serum luteinizing hormone (LH) in boys, and estradiol and LH in girls. Children were classified into pubertal stages 1 through 5 using literature-derived values [2,10].

### Energy intake

Three-day food diaries were used to assess intakes of nutrients [11]. Macronutrient intakes were determined using the USDA nutrient database [12]. Foods listed on the 3-day food diaries were labeled according to the 8-digit USDA food codes; weights of foods consumed were entered into the software; and computer programs were used to calculate the 3-day average intakes of total energy, macronutrients (carbohydrate, protein, fat) and total sugars. Intakes of added sugars were determined using the MyPyramid Equivalents Database [13]. Computer programs were used also to calculate intakes of sugars from beverages (USDA food codes were identified for all beverages including sodas, fruit-flavored drinks, fruit juice, energy/sports drinks, sweetened/flavored milks including milk shakes, tea and coffee), candy/soda-like foods (USDA food codes, 90 millions), cereal foods (USDA food codes, 50 millions), all fruit-containing foods (USDA food codes, 60 millions), fresh, dried and preserved fruit (USDA food code 60-63 & 65-69 millions), fruit beverages such as fruit juice and non-dairy fruit smoothies (USDA food code 64 millions), all dairy-containing foods (USDA food codes, 10 millions), dairy including unflavored milk, cream, whipped toppings and cheese (USDA food code 10-11.40 million; 12 & 14-19 millions), and dairy including flavored milk, milkshakes, ice cream, yogurt, and milk puddings (USDA food code 11.41-12 million; 13 & 14 millions). Non-sugar intakes were determined by difference (total carbohydrate intake minus sugar intake). Macronutrient and sugar intakes were calculated as a percentage of total energy intakes by assuming an energy value of 4 kcal/g carbohydrate and protein, and 9 kcal/g fat. Analyses were carried out following the protocol for the National Health and Nutrition Examination Survey (NHANES), with no quantification or exclusion for underreporting or over reporting.

### Statistical analysis

Statistical procedures were performed using SPSS for Windows version 16.0 (SPSS Inc, Chicago, IL). Statistical significance was defined to be p ≤ 0.05. Results with 0.05 < p < 0.10 are noted to show close associations. Differences in anthropometric indices and lipoprotein profiles in boys versus girls were performed using independent t-tests. Dixon's test for outliers was used to identify unusual values. When identified, all data for that participant were excluded from further analyses. Using Dixon's test, data for 4 of the 99 children were excluded; thus, data are presented for a total of 95 children (47 boys, 48 girls). Data were not significantly skewed for any of the variables of interest. Multiple linear regression analyses were used to assess the relationship of intake from different carbohydrate subgroups to TG, VLDL-C, IDL-C, HDL-C, HOMA-IR and ISI-FFA, after adjusting for gender, pubertal stage, waist circumference of the participating child, protein intake and fat intake. These markers were selected for further study since they were found previously to be significantly associated with total carbohydrate intake [2].

## Results

In comparison to boys, girls in this cohort had significantly higher values for measures of body fatness and were less insulin sensitive (Table 1). Carbohydrate contributed on average, nearly 50% of energy; sugars contributed ~50% of total carbohydrate consumed (Table 2) and beverages provided ~40% of total sugar.

**Table 1:** Characteristics of participating children (n = 95).

	Boys (n = 47)	Girls (n = 48)	*p*-value^a^
	Mean ± SD		
**Anthropometrics**			
Age (years)	10.4 ± 1.0	10.2 ± 1.1	*ns*
Pubertal stage (1-5)	2.6 ± 1.7	3.5 ± 1.2	0.004
Height (m)	149.3 ± 8.9	151.7 ± 9.2	*ns*
Weight (kg)	59.9 ± 18.2	66.8 ± 14.2	0.043
Body fat (%)	32.9 ± 9.5	40.2 ± 7.9	< 0.001
BMI-z score	1.91 ± 0.5	2.1 ± 0.4	0.020
WC (cm)	85.3 ± 15.4	91.0 ± 12.8	*ns*
**Biochemical parameters**			
TG (mmol/l)	66.1 ± 33.6	73.2 ± 23.4	*ns*
VLDL-C (mmol/l)	15.5 ± 4.7	16.0 ± 3.2	*ns*
IDL-C (mmol/l)	8.9 ± 4.9	9.5 ± 4.8	*ns*
HOMA-IR	1.9 ± 1.2	2.9 ± 1.4	< 0.001
ISI-FFA	0.5 ± 0.2	0.3 ± 0.2	< 0.001
**Dietary variables**			
Total energy (kcal/day)	1833 ± 684	1801 ± 619	*ns*
Carbohydrate (g/day)	218 ± 91	232 ± 87	*ns*

^a^Significance of the difference between boys and girls. ns = not statistically significant (p > 0.05).

**Table 2:** Average daily intakes of dietary components (n = 95).

Nutrients	Mean (g/day) ± SD	% of Energy intake	
		
		Mean ± SD	Range
Fat	74.0 ± 30.1	36.3 ± 5.5	21.1 - 50.0
Protein	67.0 ± 23.1	15.1 ± 3.1	8.4 - 24.9
Carbohydrate	225.0 ± 89.1	49.5 ± 7.2	36.7 - 70.7
**Carbohydrate sub groups**			
Non-sugars	120.9 ± 44.8	26.9 ± 4.7	16.6 - 39.3
Sugars	104.1 ± 54.6	22.6 ± 7.6	1.7 - 43.2
Sugars from beverages	41.1 ± 33.6	8.7 ± 5.9	0.0 - 30.0
Sugars from non-beverages	63.0 ± 34.4	13.8 ± 6.0	1.7 - 36.0
Added sugars	64.5 ± 43.2	13.7 ± 6.9	0.2 - 36.0
Non-added sugars	39.6 ± 22.1	8.9 ± 4.1	1.5 - 20.7
Sugars from Candy/soda^1^	37.0 ± 33.2	7.7 ± 6.5	0.0 - 33.3
Sugars from Cereals^2^	21.8 ± 17.8	4.7 ± 2.9	0.2 - 14.7
Sugars from all Fruit sources^3^	22.4 ± 20.6	5.0 ± 4.4	0.0 - 19.6
Sugar from fruit (no beverages)^4^	13.8 ± 14.1	3.2 ± 3.4	0.0 - 19.6
Sugar from fruit-containing beverages^5^	8.6 ± 13.9	1.9 ± 2.6	0.0 - 13.4
Sugars from all Dairy^6^	16.1 ± 15.1	3.6 ± 3.4	0.0 - 16.3
Sugars from unsweetened dairy^7^	6.2 ± 5.7	1.5 ± 1.4	0.0 - 5.8
Sugars from sweetened dairy^8^	9.8 ± 13.6	2.1 ± 2.9	0.0 - 11.5

^1 ^Candy, confections, fruit flavored drinks, sodas, syrups (USDA food code 90 Millions)

^2 ^Breakfast cereals, cakes, cookies, crackers and pastries (USDA food code 50 Millions)

^3 ^Fresh, dried and preserved fruits; fruit juice; non-dairy fruit smoothies (USDA food code 60-69 Millions)

^4 ^Fresh, dried and preserved fruit; (USDA food code 60-63 & 65-69 Millions).

^5 ^Fruit juice; non-dairy fruit smoothies (USDA food code 64 Millions).

^6 ^All dairy-containing foods including unflavored fluid milks, cream, cheese, flavored milks, milkshakes, ice cream, yogurt, and milk puddings (USDA food code 10 Millions).

^7 ^Dairy including unflavored fluid milks, cream, cheese (USDA food code 10-11.40 Million; 12 & 14-19 Millions).

^8 ^Dairy including flavored milks, milkshakes, ice cream, yogurt, and milk puddings (USDA food code 11.41-11.99 Million & 13 Millions).

Using data from a similar cohort, we previously reported [2] that higher intakes of carbohydrate (fat and protein held constant) were associated with higher TG, VLDL-C, IDL-C, HOMA-IR and lower ISI-FFA, as observed also for this cohort (Table 3 Model 1). After dividing carbohydrate intake into non-sugar versus sugar fractions (Table 3 Model 2), higher intakes of sugar was associated with significantly higher TG, VLDL-C, IDL-C, and lower ISI-FFA, and was closely associated with increased HOMA-IR. When the sugar subgroup was further divided into added sugars versus non-added (other) sugars, added sugars was associated with increased TG, VLDL-C, TG and HOMA-IR and closely associated with increased IDL-C (Table 3 Model 3).

**Table 3:** Relationship between intakes of carbohydrate sugar subgroups and cardiometabolic risk factors, assessed using five multiple linear regression models (n = 95).

	Model 1		Model 2		Model 3				
	
	Total CHO	Non sugar CHO	Sugars	Non sugar CHO	Sugars				
								
					Added sugars	Others			
**TG**	0.357*	-0.036	0.355**	0.007	0.408**	-0.062			
**VLDL-C**	0.460**	0.078	0.379**	0.090	0.335**	0.097			
**IDL-C**	0.497**	0.109	0.394**	0.085	0.239^#^	0.277*			
**HDL-C**	-0.131	-0.017	-0.111	-0.021	-0.100	-0.025			
**ISI-FFA**	-0.273*	-0.048	-0.224*	-0.020	-0.096	-0.222*			
**HOMA-IR**	0.345**	0.192	0.200^#^	0.220	0.241*	-0.054			

	**Model 4**				**Model 5**				
	
	**Non-sugar CHO**	**Sugar Source**		**Non-sugar CHO**	**Sugar Source**				
				
		**Beverage sugars**	**Other**		**Candy/soda group^1^**	**Cereal group^2^**	**Fruit group^3^**	**Dairy group^4^**	**Other**

**TG**	0.004	0.286*	0.148	0.153	0.329**	-0.069	-0.048	0.239*	0.009
**VLDL-C**	0.146	0.350**	0.108	0.237	0.294*	-0.074	0.090	0.239*	0.054
**IDL-C**	0.142	0.299*	0.185	0.251	0.275*	-0.062	0.201#	0.270*	0.239^#^
**HDL-C**	-0.041	-0.111	-0.022	-0.154	-0.020	0.017	0.056	-0.316**	-0.141
**ISI-FFA**	-0.038	-0.122	-0.160	-0.173	-0.069	0.116	-0.198*	-0.266**	-0.074
**HOMA**	0.187	0.116	0.135	0.283^#^	0.167^#^	-0.050	0.027	0.115	-0.013

*** p < 0.001, ** p < 0.01, *p < 0.05, ^# ^p = 0.05-0.10. In addition to the variables shown for each model, other variables entered simultaneously into each model included gender, pubertal stage, waist circumference, protein intake and fat intake. Values presented are standardized regression coefficients and level of significance.

^1 ^Candy, confections, fruit flavored drinks, sodas, syrups (USDA food code 90 Millions).

^2 ^Breakfast cereals, cakes, cookies, crackers and pastries (USDA food code 50 Millions).

^3 ^Fruit products including fresh, dried and preserved fruit; fruit juice; non-dairy fruit smoothies (USDA food code 60 Millions).

^4 ^Dairy including unflavored fluid milk, cream, cheese, flavored milk, milkshakes, ice cream, yogurt, and milk puddings (USDA food code 10 Millions).

In further analysis, when sugar was subdivided according to the dietary food group from which it was consumed, beverage sugar was significantly related to elevated TG, VLDL-C and IDL-C, whereas non-beverage sugar intakes were not (Table 3 Model 4). Finally, of the 9 possible groups into which foods are divided using the USDA food codes, four food groups were found to contribute 94% of the total sugar intake (Table 2). When included simultaneously in a single regression model (Table 3 Model 5), increasing consumption of sugar from the candy/soda food group was associated with highly significant increases in TG, VLDL-C and IDL-C and was closely associated with increases in HOMA-IR. Consuming sugars from cereal foods was not significantly associated with these risk factors. Intake of sugars from the total fruit group was associated with significantly lower ISI-FFA and was closely associated with increased IDL-C. Sugar intake from all dairy foods was associated with highly significant increases in TG, VLDL-C and IDL-C and with decreased HDL-C and ISI-FFA.

When the total fruit group was further divided, consumption of sugars from fruit-containing beverages was significantly associated with increased VLDL-C and IDL-C, and with decreased ISI-FFA. By contrast, sugar intakes from fresh, dried and preserved fruits did not show any significant associations with these markers (Table4 Model 6).

**Table 4:** Relationships (standardized regression coefficients and level of significance) between intakes of fruit and dairy subgroups and cardiometabolic risk factors, assessed using two multiple linear regression models (n = 95).

	Model 6				Model 7			
	
	Non-sugar CHO	Sugar Source			Non-sugar CHO	Sugar Source		
				
		Fruit (no beverages)^1^	Fruit-containing beverages^2^	Other		Dairy, un-sweetened^3^	Dairy, sweetened^4^	Other
**TG**	0.006	-0.073	0.013	0.395**	0.021	0.005	0.242*	0.249^#^
**VLDL-C**	0.096	-0.101	0.232*	0.315*	0.115	0.013	0.197^#^	0.299*
**IDL-C**	0.097	0.066	0.241*	0.281*	0.126	0.039	0.141	0.341*
**HDL-C**	-0.046	0.124	-0.026	-0.140	- 0.121	-0.097	-0.251*	0.037
**ISI-FFA**	-0.027	-0.063	-0.195*	-0.123	-0.098	-0.047	-0.171*	-0.139
**HOMA**	0.203	0.012	0.027	0.198^#^	0.213	-0.027	0.123	0.153

*** p < 0.001, ** p < 0.01, *p < 0.05, ^# ^p = 0.05-0.10. In addition to the variables shown for each model, other variables entered simultaneously into each model included gender, pubertal stage, waist circumference, protein intake and fat intake. Values presented are standardized regression coefficients and level of significance.

^1 ^Fresh, dried and preserved fruit; (USDA food code 60-63 & 65-69 Millions).

^2 ^Fruit-containing beverages including fruit juice, non-dairy fruit smoothies (USDA food code 64 Millions).

^3 ^Dairy including unflavored milk, cream (including whipped toppings) and cheese (USDA food code 10-11.40 Million; 12 & 14-19 Millions).

^4 ^Dairy including flavored milk, milkshakes, ice cream, yogurt, and milk puddings (USDA food code 11.41-11.99 Million & 13 Millions).

When the total dairy group was sub-divided, sugar intakes from sweetened dairy products showed significant association with increased TG and with decreased HDL-C and ISI-FFA, whereas sugar intake from unsweetened dairy foods did not (Table 4 Model 7).

## Discussion

Our main outcomes highlight the association of intakes of carbohydrate from different subgroups with key markers of cardiometabolic risk in high BMI AA children. Importantly, the variance in TG, VLDL-C, IDL-C and ISI-FFA contributed by total carbohydrate appeared to be mainly from sugars, suggesting that sugar fractions contributed to the undesirable effects of increasing total carbohydrate intake. Thus, increases in total carbohydrate intake, due to increased sugar intake, were associated with undesirable increases in several classes of plasma lipids. Additionally, sugar intake was closely associated with decreases in HOMA-IR. (Table 3 Model 2).

New evidence on the relationship between intake of sugars and cardiovascular health has emerged since the last American Heart Association (AHA) scientific statement was published in 2002 [14]. In 2006, the AHA revised their diet and lifestyle recommendations, adding a recommendation to minimize intakes of beverages and foods with added sugars [15]. Other recent findings have suggested that higher consumption of added sweeteners such as high fructose corn syrup can lead to weight gain, increased insulin resistance, a lowering of HDL-C, and an increase in triglyceride levels [16,17]. In our study, added sugars intake was associated with increased TG, VLDL-C & HOMA-IR (Table 3 Model 3), suggesting that added sugars have undesirable effects in children similar to those in adolescents [18] and adults [15].

Our results are also consistent with AHA's recent statement that high intake of added sugars in the setting of a worldwide pandemic of obesity and cardiovascular disease have heightened concerns about the adverse effects of excessive consumption of added sugars [19], suggesting that these recommendations made for adults regarding sugar intake may also apply to children.

Results from the Framingham Heart Study suggest that soft drink consumption is associated with a higher prevalence and incidence of multiple metabolic risk factors in middle-aged adults [20]. Many clinical studies have linked the rising consumption of soft drinks to the present epidemic of obesity and diabetes mellitus among children and adolescents [21-23]. In contrast, Vanselow et al. recently reported that, with the exception of low-calorie soft drinks, intakes of calorie-containing beverages were not associated with change in BMI in adolescents [24]. In our study, we were not able to perform a regression analysis with soft drinks as the dependent variable, since 51% of our sample did not consume sodas during the 3-day diet recording period. However, the aggregate of sugar intakes from all beverages were significantly associated with TG, VLDL-C and IDL-C (Table 3 Model 4). Additionally, in our study, higher intakes of sugars from the food group that included candy, confections, fruit flavored drinks, sodas and syrups were significantly associated with elevated TG, VLDL-C and IDL-C (Table 3 Model 5). Intake of sugars from the all fruit group (this includes fresh fruit, processed fruit and fruit juice) was associated with reduced adipocyte insulin sensitivity and was closely associated with IDL-C (Table 3 Model 5). The all dairy products group (this includes processed dairy foods such as fruit smoothies, ice cream, milk and flavored milk, yogurt) was associated with elevated TG, VLDL-C and IDL-C and reduced HDL-C and adipocyte insulin sensitivity in these children (Table 3 Model 5).

In our study, sugar intakes from the dairy, and to a lesser extent, the fruit food groups were associated with increased cardiometabolic risk factors in these high-BMI children. When these food groups were further divided, this risk was associated with sugars in fruit-containing beverages and with sweetened dairy foods (Table 4). Thus, our results support AHA's diet and lifestyle recommendations, to minimize intakes of beverages and foods with added sugars [15]. As no positive association was observed between intakes of non-beverage fruit-containing foods and these risk factors, this strengthens the recommendations for increasing fresh fruit consumption over beverages in children. Similarly, "healthy", unsweetened dairy foods were not significantly associated with increased risk, nor were they were associated with reduction in risk factors. Thus, recommendations that these children increase dairy intakes as a means of improving bone health should focus on the unsweetened dairy foods, and not on sweetened dairy.

Limitations of this study include restriction to low-income, inner-city, African American children and exclusion of children with BMI's less than the 85^th ^percentile when matched for age and gender. These limitations preclude comparisons among children of different races, ages and socioeconomic backgrounds, and comparisons with lower BMI children. The limitations inherent in collecting dietary data, regardless of population, are also recognized. This is a cross-sectional analysis of data, precluding a cause and effect relationship. Future longitudinal studies, with measurements at several time-points, would be needed to evaluate a causal relationship. Also, replications in longitudinal studies with larger sample sizes, and in multiracial cohorts are warranted.

## Conclusion

Based on our analysis, we conclude that increases in carbohydrate energy in the form of sugars were associated with undesirable increases in several classes of plasma lipids and with decreases in both hepatic glucose and adipocyte fatty acid insulin sensitivity. Higher intakes of sugars from the candy/soda food group, from fruit-containing beverages, and from sweetened dairy foods were associated with increases in several cardiometabolic risk factors. This analysis suggests that increases in many types and sources of sugar may be detrimental to cardiometabolic health in high BMI children.

## Abbreviations

AA: African American; BMI: Body mass index; CVD: Cardio vascular disease; HOMA-IR: Homeostasis model assessment of insulin resistance; IDL-C: Intermediate density lipoprotein cholesterol; ISI-FFA: Fatty acid insulin sensitivity; NEFA: Non-esterified fatty acids; TG: Triglyceride; VLDL-C: Very low density lipoprotein cholesterol; HDL-C: High density lipoprotein cholesterol; WC: Waist circumference.

## Conflict of interests

The authors declare that they have no competing interests.

## Authors' contributions

Contributor's list: SS contributed in statistical analysis, preparation of the manuscript and submission. LSR participated in the development of the protocol, analytical framework for the study and patient screening. RHL provided expertise as a pediatric endocrinologist and child health specialist. SEF was the principal investigator of the study. She supervised the design and execution of the study and manuscript. All authors have read and approved the final manuscript.
